# Celastrol Attenuates Lipid Accumulation and Stemness of Clear Cell Renal Cell Carcinoma via CAV-1/LOX-1 Pathway

**DOI:** 10.3389/fphar.2021.658092

**Published:** 2021-04-16

**Authors:** Chan-Juan Zhang, Neng Zhu, Yu-Xiang Wang, Le-Ping Liu, Tan-Jun Zhao, Hong-Tao Wu, Duan-Fang Liao, Li Qin

**Affiliations:** ^1^ Division of Stem Cell Regulation and Application, Department of Pharmacology, School of Pharmacy, Hunan University of Chinese Medicine, Changsha, China; ^2^ Department of Urology, The First Hospital of Hunan University of Chinese Medicine, Changsha, China; ^3^ Institute of Innovation and Applied Research in Chinese Medicine, Hunan University of Chinese Medicine, Changsha, China; ^4^ Department of Urology, The Second Xiangya Hospital of Central South University, Changsha, China

**Keywords:** celastrol, clear cell renal cell carcinoma, lipid accumulation, stemness, cav-1, LOX-1

## Abstract

Clear cell renal cell carcinoma (ccRCC) is characterized by abnormal lipid accumulation. Celastrol is a pentacyclic triterpene extracted from Tripterygium wilfordii Hook F with anti-cancer activity. In the present study, the anticancer effects of celastrol on ccRCC and the underlying mechanisms were studied. Patients with reduced high density lipoprotein (HDL) and elevated levels of triglyceride (TG), total cholesterol (TC), low density lipoprotein (LDL) was found to have higher risk of ccRCC. In ccRCC clinical samples and cell lines, caveolin-1 (CAV-1) was highly expressed. CAV-1 was identified as a potential prognostic biomarker for ccRCC. Celastrol inhibited tumor growth and decreased lipid deposition promoted by high-fat diet *in vivo*. Celastrol reduced lipid accumulation and caveolae abundance, inhibited the binding of CAV-1 and lectin-like oxidized low-density lipoprotein receptor-1 (LOX-1) in ccRCC cells. Furthermore, celastrol attenuated stemness through blocking Wnt/β-catenin pathway after knockdown of CAV-1 and LOX-1. Therefore, the findings suggest that celastrol may be a promising active ingredient from traditional Chinese medicine for anti-cancer therapy.

## Introduction

Clear cell renal cell carcinoma (ccRCC) is the most lethal and the third most common urological disease, accounting for approximately 80% of diagnosed RCCs (Miller et al., 2019). ccRCC is also considered to be a chronic metabolic disease that exhibits abnormal lipid accumulation in the cytoplasm (Hakimi et al., 2016). Aberrant metabolisms such as glucose metabolism and lipid metabolism, especial lipid uptake and transportation, has been recognized in cancer cells to facilitate cell growth and proliferation (Yue et al., 2014). Excess lipids in cancer cells are stored in lipid droplets, and high levels of lipid content are currently considered to be a hallmark of cancer aggressiveness (Currie et al., 2013). Whether parameters related to the metabolic processes during the progression of ccRCC could be potential diagnostic biomarkers and therapeutic targets is still to be addressed.

Understanding the mechanisms by which lipid contributes to tumor growth will lead to innovative approach for treating cancer. Caveolae are 50–100 nm flask-shaped or cup-shaped invaginations of the plasma membrane enriched in cholesterol and glycosphingolipids (Gervásio et al., 2011). Caveolin-1 (CAV-1) acts as an essential structural protein for the formation of caveolae. CAV-1 directly binds to cholesterol with high affinity and serves as the site for cholesterol uptake from the extracellular environment (Murata et al., 1995). Several observations support the role of CAV-1 in maintaining lipid homeostasis, tumorigenesis, and signal transduction (Fernández-Rojo et al., 2013; Mao et al., 2019). CAV-1 plays dual effects as an oncogene or as a tumor suppressor. Although it has been suggested that increased CAV-1 expression is related to ccRCC and contributes to the progression of ccRCC, the precise role of CAV-1 remains controversial.

Recent study has reported that lectin-like oxidized low-density lipoprotein receptor-1 (LOX-1) receptors are distributed within lipid rafts in caveolin-enriched membranes (Li et al., 2017). LOX-1 was first identified as a major receptor of oxidized low-density lipoprotein (ox-LDL) and is mainly expressed in endothelial cells, smooth muscle cells, macrophages, and monocytes (Yan et al., 2011). Strikingly, the expression of LOX-1 has been demonstrated to be strongly implicated in carcinogenesis (Balzan and Lubrano 2018). Activation of LOX-1 promotes the growth and metastasis of gastric cancer (Li et al., 2017). Moreover, highly expressed LOX-1 is a significant prognosis factor of tumor progression in advanced-stage of prostate cancer and colorectal cancer (González-Chavarría et al., 2018; Nakashima-Nakasuga et al., 2020).

Stemness has been characterized in many cancers and implicated in tumor recurrence and metastasis. The Wnt pathway activates a broad spectrum of downstream targets through two independent branches mediated by β-catenin (defined as the canonical pathway) or non-canonical pathway, respectively (Xue et al., 2016). Wnt/β-catenin pathway usually contributes to cancer cell proliferation, metabolism, and stem cell renewal, etc. CAV-1 prevents gastric cancer cells from the cisplatin-induced apoptosis by activating the Wnt/β-catenin signaling pathway (Pai et al., 2017). In addition, our previous study has revealed that Wnt5a promotes stemness characteristics, leading to the tumorigenesis and metastasis of nasopharyngeal carcinoma cells (Qin et al., 2015). Therefore, a detailed understanding of these mechanisms and agents that regulate lipid metabolism and stemness may be an effective strategy for the treatment of cancers.

Here we report celastrol, a pentacyclic triterpene extracted from the traditional Chinese medicine *Tripterygium wilfordii* Hook F. It is worth noting that celastrol is listed by the journal Cell as one of the five traditional medicines most likely to be transformed into modern drug (Corson and Crews 2007). Celastrol is also identified as a leptin sensitizer and a potential anti-obesity therapeutic agent (Liu et al., 2015). Due to the reduction of hepatic steatosis and the accumulation of intrahepatic triglycerides, celastrol-treated mice exhibited a decrease in liver weights. Celastrol is a potential anti-cancer agent related to a variety of molecular mechanisms, such as anti-proliferation, anti-metastasis, pro-apoptosis, and anti-angiogenesis (Kashyap et al., 2018). Recently report found that celastrol attenuated stemness-like properties of ovarian cancer SKOV3 cells (Li et al., 2019). In the present study, we aimed to investigate the antitumor effects and possible mechanisms of celastrol on ccRCC both *in vitro* and *in vivo*. Considering the contribution of lipid disorder to ccRCC progression and the effect of celastrol on lipid metabolism, we proposed that celastrol might be beneficial agent against stemness characteristics of ccRCC related to lipid abnormalities.

## Methods

### Human ccRCC Clinical Information and Specimens

We retrospectively studied the prospective kidney cancer database of the second Xiangya Hospital of Central South University, including follow-up data of 362 consecutive patients from 2011 to 2017. A total of 4 ccRCC patient samples were obtained from the second Xiangya Hospital of Central South University between 2017 and 2019. The samples included both cancer tissue and adjacent normal tissue (1 cm away from the margin of the tumor tissues). All patients included in the study had no history of adjuvant therapy (chemotherapy or radiotherapy).

### RNA Sequencing

Total RNA was extracted from human ccRCC and patient-matched normal tissues with TRIzol (Invitrogen, Thermo Fisher Scientific, Inc. United States) according to the manufacturer’s protocol. RNA quality was examined by gel electrophoresis, and only paired RNA of high quality was used for RNA sequencing. RNA sequencing libraries were prepared according to the manufacturer's instructions and then sequenced with the Illumina HiSeq 2000 at Riobo company (Guangzhou).

### Bioinformatics Analysis

We obtained the standardized mRNA expression of CAV-1 in ccRCC and disease-free survival time (DFS) from the TCGA database and GTEx database.

### Western Blotting

Cultured cells, clinical sample or xenograft tumor tissues were extracted from radio-immunoprecipitation assay (RIPA, Cwbiotech, China) buffer and protease inhibitor cocktail (Cwbiotech, China). Protein concentrations were determined by the BCA™ Protein Assay Kit according to the protocol provided by the manufacturer (Cwbiotech, China). Protein (50 μg) loaded in each lane was subjected to 10% sodium dodecyl sulfate-polyacrylamide gel electrophoresis (SDS-PAGE), and then transferred to a polyvinylidene difluoride (PVDF, Merck Millipore, Billerica, MA, United States) membrane. After blocking with 5% skimmed milk for 2 h, PVDF membranes were incubated with a primary antibody overnight at 4°C, and then incubated with a secondary antibody for 1.5 h. The protein bands were detected using visualizer (Tanon, China).

### Cell Culture

Human ccRCC cell lines (786-O, A498, SN12C and OS-RC-2) and renal epithelial cells (HK-2 cell) were obtained from the Cell Bank of the Chinese Academy of Sciences (Shanghai, China). ccRCC cells were cultured in RPMI1640 (Gibco, United States) supplemented with 10% fetal bovine serum (Gibco, Grand Island, NY, USA) and 1% streptomycin-penicillin (Gibco, Grand Island, NY, United States). Dulbecco’s modified Eagle’s medium (DMEM, Gibco, Grand Island, NY, United States) containing 10% fetal bovine serum (FBS; Gibco, Grand Island, NY, United States) and 1% streptomycin-penicillin were used for HK-2 culture. All cells were maintained at 37°C in a humidified atmosphere with 5% CO2.

### Quantitative Real Time-PCR

The total RNA was isolated using the TRIzol isolation methods following the manufacturers’ recommendations. The RNA concentrations were quantified using Nanodrop 2000 spectrophotometer (Thermo Fisher Scientific, Waltham, MA, United States). The mRNA expression was calculated based on the 2^−ΔΔ^Cq method. qPCR was conducted using an Applied BioRad CFX96 Real-Time PCR. The primers used for qPCR are available on request.

### Animal Experiment

Male BALB/c nude mice (Slac, SCXK (Xiang)2014-0002, 4 weeks old) were kept in a specific pathogen-free animal laboratory. The animal study was reviewed and approved by Hunan University of Chinese Medicine. BALB/c nude mice were originated from the mus musculus. The two main defects of BALB/c nude mice are failure of hair growth and hypoplasia of thymic epithelium due to developmental failure of the thymic anlage. BALB/c nude mice also have poor response to thymus-dependent antigens because of the defect in helper T-cell activity. For subcutaneous xenograft models, approximately 1.0 × 10^7^ 786-O cells were subcutaneously into the left flank of mice, which were randomly assigned to 6 groups (*n* = 3) by using blinded allocation. BALB/c nude mice without tumorigenesis were excluded. BALB/c nude mice fed with a normal diet (control group) and high-fat diet (HFD group). The groups treated with CeT were fed with HFD. Body weight of mice and tumor volumes were measured every two days. Tumor volumes were recorded at the indicated time points by measuring the tumors with calipers and calculating the volumes with the formula L×W^2^/2 (L is the length of the longer axis and W is the length of the shorter axis). Four weeks after the injection, all mice were euthanized under anesthesia with isoflurane inhalation (2%), and the tumors were excised, weighed, and photographed. Some tumors were stored at −80°C for western blot analysis, others were fixed in 4% paraformaldehyde for staining experiment.

### Hematoxylin-Eosin (H&E) Staining

Tissues were fixed in 4% paraformaldehyde and processed for histologic examination, including embedding in paraffin, sectioning, and staining with hematoxylin and eosin (H&E). The formaldehyde-fixed epithelial tissues were dehydrated using gradient ethanol (70%, 80%, 90%, 95%, 100%, 5 min each), cleared twice with xylene (10 min each), embedded with paraffin, and subsequently cut into 4 μm serial sections. The sections were baked at 60 °C for 1 h and dewaxed with xylene. Following the removal of xylene using gradient ethanol, the sections were washed, stained with hematoxylin for 10 min, and washed with distilled water for a 1 min period. After being differentiated with 1% hydrochloric ethanol for 20 s, the sections were washed with distilled water for 1 min and turned blue using 1% ammonia for 30 s. The sections were then additionally stained with eosin for 3 min, dehydrated with gradient ethanol (2 min for each), cleared twice with xylene (5 min for each), and mounted using neutral balsam. Then the samples were observed under an optical microscope (×40) (Olympus, Tokyo, Japan).

### Plasma Biochemical Analysis

Blood was collected after 4 weeks of treatment with the corresponding medicine into heparinized tubes. Biochemical parameters, including plasma high density lipoprotein (HDL), triglyceride (TG), total cholesterol (TC), low density lipoprotein (LDL), and very low density lipoprotein (VLDL) levels, were measured using an autoanalyzer (Beckman Coulter, Miami, FL, USA).

### Oil Red O Staining Assay

Freeze tumors were cryo-sectioned using Leica CM3050 cryotome (Leica Biosystems Inc., Wetzlar, Germany) and 7 μm sections were obtained. The slides were placed to 25°С and washed in running water to remove the optimal cutting temperature (OCT) compound (Tissue-Tek, Torrance, CA, United States). Slides were placed in 50% isopropanol for 3 min and in 100% isopropanol for 3 min and stained with 0.5% Oil red O (Solarbio, China) in 100% isopropanol for 2 h. Then, slides were differentiated in 85% isopropanol for 3 min three times, washed with running water, and stained with Mayer’s hematoxylin for 15 s followed by bluing in running water for 10 min. Slides were mounted with Glycerol Jelly Mounting Medium (Beyotime, China) before analyzed.

### Immunohistochemistry (IHC)

Tissue samples collected from ccRCC patients and tumor xenograft mice were made into 4% paraformaldehyde blocks using the following steps: formalin fixation, dehydration and paraffin embedding. The sections were incubated overnight at 4°С with each primary antibody and incubated for 1 h at 25°С with secondary antibodies. Proteins were visualized using EnVision Detection Systems (Dako Japan, Tokyo, Japan). Immunochemical results were evaluated by an experienced pathologist with a semiquantitative approach assigning an H-score (or “Histoscore”) to the tumor sample. The percentage of tumor cells was determined for each different nuclear staining intensity (0/+/++/+++), and the sum of the individual H-scores for each intensity level was then calculated using the following equation: H-score = 1 × (% of cells with an intensity of 1+) + 2 × (% of cells with an intensity of 2+) + 3 × (% of cells with an intensity of 3+).

### Cell Counting Kit-8 Assay (CCK-8)

Cell viability was evaluated using the CCK-8 kit (Beyotime, China) according to the manufacturer's instructions. Cells were seeded in 96-well flat bottom microtiter plates at a density of 5 × 10^3^ cells per well. The absorbance was measured on a microplate reader (Synergy HT, Bio-Tek, Biotek Winooski, Vermont, United States) at 450 nm.

### BODIPY-Cholesterol Staining

We examined cholesterol efflux by using fluorescent sterol, boron dipyrromethene difluoride linked to sterol carbon-24 (BODIPY-cholesterol; No. GC42964, Glpbio, Montclair, CA, United States). 786-O cells were cultured in 6-well plates, and then cultured in serum-free medium containing 0.1 ml labeling media for 1 h. The cells were washed three times with MEM-HEPES (Gibco, Grand Island, NY, United States), and then cultured in serum-free medium containing treatment factors for 24 h. Fluorescent intensity was determined by a fluorescence microscope unit with ×40 magnification and photographed with OLYMPUS Stream system (Olympus DP73, TH4-200, Tokyo, Japan). Fluorescent intensity was quantified from at least 3 random fields per slide, from three slides per experimental condition and graphed.

### Cholesterol Enzyme Assay

Cholesterol content was determined by enzymatic assay kit (Applygene Technologies Inc., China). Cholesterol standard solution was appropriately diluted using cholesterol assay buffer. Detection reagent was added to 50 μl of cholesterol standard or collected cell lysate supernatant for 5 min. Optical density values at 535/590 nm were measured. Standard curve was prepared using cholesterol standard solution, and the concentrations of cholesterol were calculated according to the standard curve.

### Immunofluorescence Staining

Cultured cells were fixed by 4% paraformaldehyde, treated with 0.1% Triton X-100 (Sigma-Aldrich, St. Louis, MO, United States) for 5 min and blocked in 3% bovine serum albumin (BSA; Solarbio, China) for 1 h. The cells were then incubated with primary antibodies with a dilution of 1:100 overnight at 4°C. After incubation with fluorescent-dye-conjugated secondary antibodies and DAPI. Immunofluorescent microscopic images of the cells were obtained and viewed by using an inverted fluorescence microscope (Olympus DP73, TH4-200, Tokyo, Japan). Quantitative analysis of fluorescence intensity was performed with Image Pro Plus 6.0 software (Media Cybernetics, United States).

### Sphere Formation Assay

The 786-O cells were plated at a density of 5 × 10^3^ cells per well in ultra-low attachment six well plates (Corning Incorporated, Corning, NY, United States) and incubated in DMEF/F12 (1:1) supplemented with B-27 (Gibco, Grand Island, NY, United States), 10 ng/ml fibroblast growth factor-basic (bFGF; Gibco, Grand Island, NY, United States) and 10 ng/ml epidermal growth factor (EGF; Gibco, Grand Island, NY, United States). 786-O cells were treated with corresponding agent after plating for 24 h. Every three days, half of the medium was replaced. Cells were cultured for 10 days. The generated spheroids were counted and photographed using inverted microscope (Olympus DP73, TH4-200, Tokyo, Japan). The number of spheres with a diameter of >50 μm was quantified by ImageJ software.

### Small Interfering RNA (SiRNA) Transfection

Three double-stranded siRNAs targeting CAV-1 or LOX-1 and a negative control siRNA were designed and synthesized by Ribobio Company (China) and transfected into 786-O cells using riboFECT™ CP Reagent (Ribobio, China) for 24 h following the manufacturer’s guidelines. CAV-1-01, CAV-1-02 and CAV-1-03 correspond to 3 different siRNA against CAV-1. LOX-1-01, LOX-1-02 and LOX-1-03 correspond to 3 different siRNA against LOX-1.

### Transmission Electron Microscopy (TEM)

The 786-O cells were fixed in 1.6% glutaraldehyde prior to post-fixation in osmium tetroxide and uranyl acetate *en bloc* staining. Samples were processed and embedded in Spurr epoxy resin, thin sectioned, and counterstained with lead citrate. Digital images were obtained with a Hitachi HT7700 TEM (Hitachi, Tokyo, Japan).

### Co-Immunoprecipitation

The 786-O cells were lysed on ice in 20 mM Tris-HCl (pH 7.4), 1% Triton X-100, 0.025% SDS, 100 mM NaCl, 1 mM Na_3_VO_4_, 10 mM NaF, and 1% protease inhibitor cocktail (Sigma, United States). Soluble extracts were incubated for 2 h at 4°C with relevant antibodies: anti-CAV-1 (Abcam, United States), anti-LOX-1 (Proteintech, United States) and a negative isotype control mouse immunoglobulin (IgG) (Santa Cruz Biotechnology, United States). Immune complexes were precipitated with protein A/G agarose (Santa Cruz Biotechnology, United States). Western blotting was performed as described before.

### Statistical Analysis

All values are expressed as mean ± SDs. All experiments were independently repeated at least three times. A paired *t*-test was used to compare patient tissues. Student’s t test was used in the two groups of experiments. One-way analysis of variance (ANOVA) ANOVA with Tukey’s post-test (One-way ANOVA for comparisons between groups) were used to compare values among different experimental groups. All statistical analyses were carried out using GraphPad Prism software (La Jolla, CA, United States). The statistical significance was set at *p* < 0.05.

## Results

### The Correlation of the Clinical Characteristics and Lipid Profile in ccRCC Patients

A total of 362 ccRCC patients were included in this retrospective investigation. The detailed clinicopathological data of the patients are shown in Table 1. The patients included 245 men and 117 women, with a median age of 53 years (range: 2–88 years). Among the surgical patients, 29 cases had lymph node metastasis and 27 cases had distant metastasis (Table 1). Of these patients, 168 cases had HDL levels <1.04 mmol/L, 180 cases had TG levels >1.21 mmol/L, and 60 cases had high levels of TC (>5.89 mmol/L). There were 108 cases with LDL level >3.4 mmol/L (Table 1). These data indicated a positive correlation between lipoproteins and ccRCC risk.

**TABLE 1:** Clinicopathological characteristics of ccRCC patients.

Characteristics		Cases	Patients (%)	Characteristics		Cases	Patients (%)
Age (years)	<53	172	47.5	Lymph node metastasis		29	8.0
	≥53	190	52.5	Distant metastasis		27	7.5
Gender	Male	245	67.7	HDL (mmol/L)	<1.04	168	46.4
	Female	117	32.3		1.04–1.55	60	16.6
Tumor size (cm)	>3	142	39.2		>1.55	24	6.6
	≤3	55	15.2	TG (mmol/L)	0.22–1.21	36	9.9
Tumor number	>1	42	11.6		>1.21	180	49.7
	= 1	146	40.3	TC (mmol/L)	<2.86	36	9.9
T Stage	T1+T2	42	11.6		2.86–5.89	84	23.2
	T3+T4	6	1.66		>5.89	60	16.6
N stage	N0	40	11.0	LDL (mmol/L)	0–3.4	72	19.9
	N1	5	1.38		>3.4	108	29.8
M Stage	M0	42	11.6				
	M1	5	1.38				

CcRCC, Clear cell renal cell carcinoma; HDL, high density lipoprotein; TG, triglyceride; TC, total cholesterol; LDL, low density lipoprotein.

### Identification of CAV-1 as a Potential Biomarker of ccRCC

**FIGURE 1:**
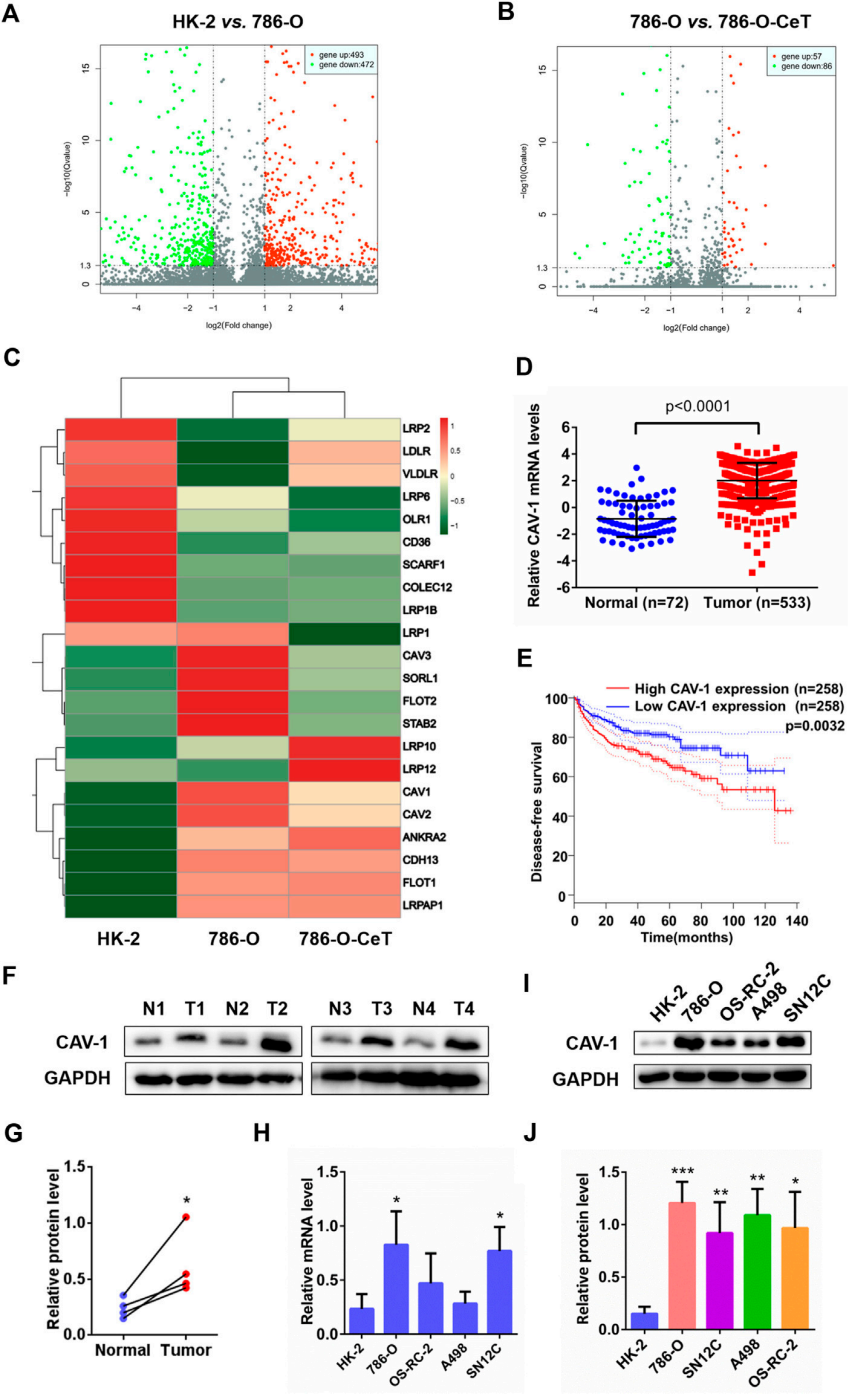
Microarray analysis showed differentially expressed genes in regulating lipid uptake. CAV-1 was highly expressed in ccRCC tissues and cell lines **(A)** Volcano plot of *p* values as fold change for mRNAs in 786-O cells and HK-2 cells **(B)** The volcano plot displayed different genes in celastrol (CeT)-treated 786-O cells vs. untreated 786-O cells. The *x*-axis was the log2 ratio of gene expression between two stages; the *y*-axis was adjusted the q value based on -log10. The red dots represented genes that were significantly upregulated, and green dots represented genes that were dramatically downregulated **(C)** Heat map of lipid uptake-related genes in HK-2 cells, untreated 786-O cells and CeT-treated 786-O cells **(D)** The mRNA level of CAV-1 was obtained from TCGA_KIRC datasets and GTEx datasets **(E)** The correlation between CAV-1 expression and disease-free survival time of ccRCC patients was analyzed by Kaplan-Meier **(F,G)** Western blotting of CAV-1 expression in ccRCC tissues (T) and adjacent tissues (N) **(H–J)** qRT-PCR analysis and western blotting were used to detect the mRNA and protein expression of CAV-1 in HK-2 cells and ccRCC cell lines (786-O, OS-RC-2, A498, SN12C). The data were shown as the mean ± SD of three replicates. ^*^ *p* < 0.05, ^**^ *p* < 0.01, ^***^ *p* < 0.001 vs. the corresponding control.

Microarray gene expression analysis revealed differentially expressed genes in HK-2 cells, 786-O cells and celastrol-treated 786-O cells. We first filtered genes by applying a change of ≥ 1-fold as a cut-off value to identify genes that are significantly up-and down-regulated (*p* < 0.05). Comparing with HK-2 cells, 493 genes were upregulated, and 472 genes were downregulated in 786-O cells (Figure 1A). The gene expression of 786-O cells in response to celastrol treatment were evaluated. There were 143 differentially expressed genes identified in 786-O cells with celastrol treatment, including 57 up-regulated and 86 down-regulated genes (Figure 1B). Then, lipid uptake-associated genes were examined, and the results were plotted in the heat map (Figure 1C). Among lipid uptake genes, CAV-1 was specifically up-regulated in 786-O cells and down-regulated by celastrol. Then, the mRNA expression of CAV-1 in ccRCC tissues and adjacent tissues from publicly available TCGA-KIRC database and GTEx-KIRC database were assessed. Bioinformatics analysis showed that the expression of CAV-1 in ccRCC tissues was increased compared with that in adjacent tissues (Figure 1D). Then, the prognostic level of CAV-1 in ccRCC was determined. The disease-free survival time of the high CAV-1 expression group was lower than that of the low CAV-1 expression group (Figure 1E). Consistently, the protein expression of CAV-1 in ccRCC tissues was remarkably increased relative to adjacent tissues (Figures 1F,G). The mRNA and protein levels of CAV-1 in ccRCC cell were also significantly higher than that in HK-2 cells, especially in 786-O cells (Figures 1H–J). These findings indicated that CAV-1 might be a potential biomarker for ccRCC diagnosis.

### Celastrol Inhibited Tumor Growth of ccRCC *in vivo*

**FIGURE 2:**
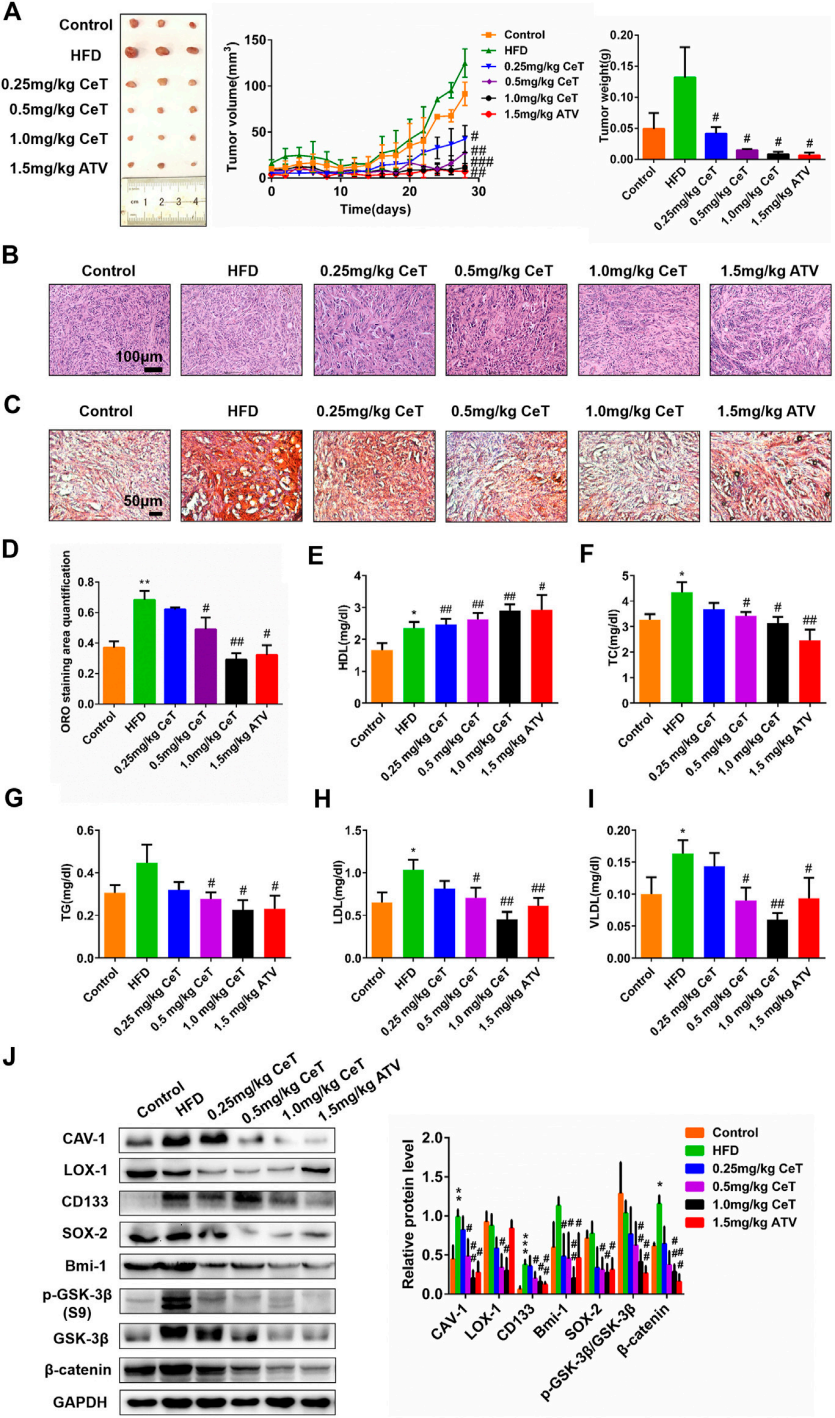
Celastrol inhibited the growth of xenografts tumors of 786-O cells *in vivo* **(A)** Nude mice were injected subcutaneously with 1 × 10^7^ 786-O cells. Mice were then treated with CeT (0.25, 0.5, 1.0 mg/kg), ATV (1.5 mg/kg) for 4 weeks. The tumor volume at the indicated time-points were calculated every two days after treatment, and the tumor weight was calculated at end of the experiments **(B)** Representative images of H&E staining. Scale bar: 100 μm **(C, D)** Lipid deposition of tumor tissues was measured by Oil red O staining. Scale bar: 20 μm. **(E-I)** The biochemical analysis was used to evaluate plasma levels of HDL, TC, TG, LDL, and VLDL. **(J)** The protein levels of CAV-1, LOX-1, CD133, Bmi-1, SOX-2, p-GSK-3β (S9), GSK-3β, and β-catenin in xenografts tumors were analyzed by western blotting. The data were shown as the mean ± SD of three replicates. ^*^ *p* < 0.05, ^**^ *p* < 0.01 vs. the control group; ^#^ *p* < 0.05, ^##^ *p* < 0.01, ^###^ *p* < 0.001 vs. the HFD group.

To assess the effect of celastrol in ccRCC *in vivo*, the tumor xenograft model by subcutaneous injection of 786-O cells was employed. As shown in Figure 2A, nude mice fed with HFD promoted tumor growth, while celastrol attenuated tumor growth rate in a dose-dependent manner. Tumor weight were higher in the HFD group, whereas significantly lower in the celastrol group and atorvastatin group (Figure 2A). Atorvastatin is the most commonly used anti-cholesterol drug and has been reported to inhibit the progression of various cancers, including RCC. The body weight of mice was monitored throughout the whole study, and the results showed that the HFD made the mice get heavier, but their body weight was dose-dependently decreased after each exposure to celastrol (Figure 2A). Atorvastatin at 1.5 mg/kg kept the body weight steady of nude mice even fed with HFD (Supplementary Figure S1A). Subsequently, the results of H&E staining showed that tumor xenograft model fed with normal diet exhibited cylindrical shape, large nuclei, increased nuclear/cytoplasmic ratio and cellular cleavage, as well as the glands with abnormal sizes and shapes (Figure 2B). The tumor xenograft model fed with HFD showed that the cancer was poorly differentiated and invaded into the mucous membrane, accompanied by degeneration and necrosis of crypt cells and an amount of infiltrative inflammation (Figure 2B). Celastrol significantly relieved these symptoms, only showing moderate edema in lamina propria interstitial or mild inflammatory cell infiltration (Figure 2B), and similar results were observed in atorvastatin group (Figure 2B). The lipid accumulation was increased in the high-fat diet-fed animal model and celastrol significantly reduced lipid deposition in tumor tissues (Figures 2C,D). Biochemical analysis showed that celastrol increased HDL level, while reduced plasma levels of TC, TG, LDL and VLDL in nude mice fed HFD (Figures 2E–I). In addition, the tumor resected from the HFD group expressed higher levels of CAV-1, LOX-1, CD133, Bmi-1, SOX-2, p-GSK-3β (S9)/GSK-3β and β-catenin than the tumor from the control group, while celastrol down-regulated the levels of these proteins (Figure 2J). The protein levels of these molecules were also confirmed by IHC (Supplementary Figures S1B–I). These results indicated that celastrol inhibited tumor growth of ccRCC in mice fed with high-fat diet through reducing lipid deposition and stemness proteins expression.

### Celastrol Inhibited Lipid Accumulation and Stemness Characteristics of ccRCC Cells

**FIGURE 3:**
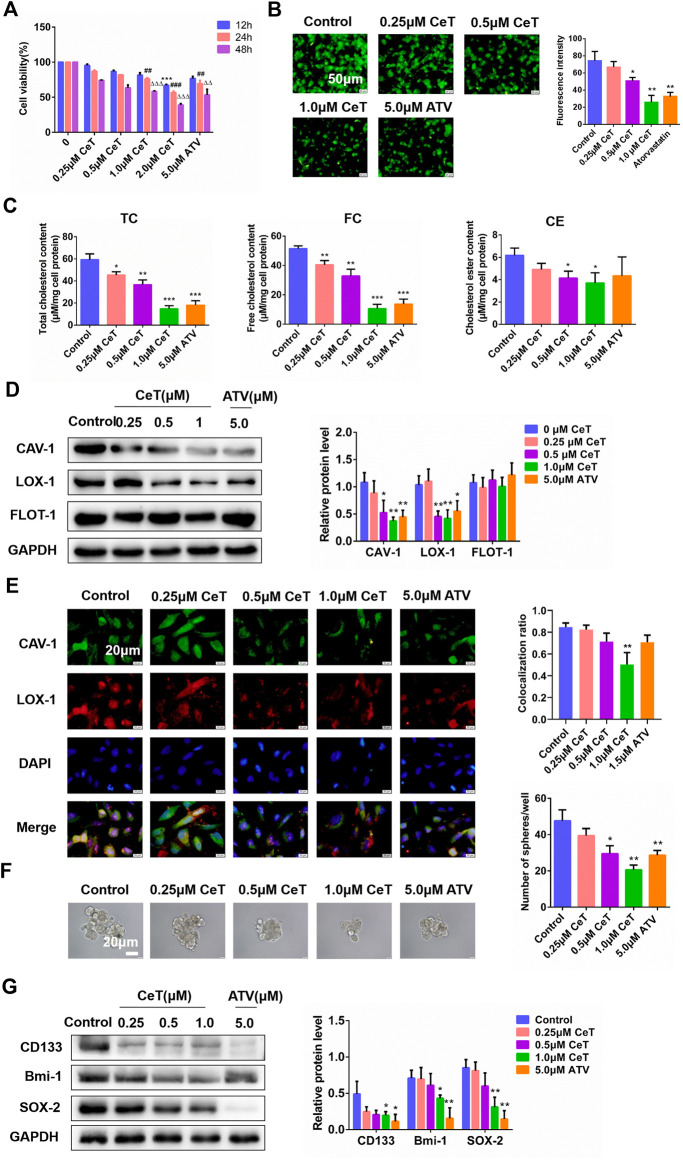
Celastrol attenuated lipid accumulation and suppressed stemness characteristics of 786-O cells **(A)** The cytotoxicity of CeT (0.25, 0.5, 1.0, 1.5 2.0 μM) on 786-O cells was determined by CCK-8 assay. 12 h: ***p < 0.001 vs. control. 24 h: ^##^ *p* < 0.01, ^###^ *p* < 0.001 vs. control. 48 h: ^△△^ *p* < 0.01, ^△△△^ *p* < 0.001 vs. the control **(B)** Fluorescence microscopy of 786-O cells stained with BODIPY 493/503. Scale bar: 50 µm **(C)** Quantification of levels of intracellular TC, FC, and CE **(D)** The expression of CAV-1, LOX-1, FLOT-1 was verified by western blotting **(E)** The co-localization of CAV-1 (green) and LOX-1 (red) in 786-O cells was confirmed by immunofluorescence staining. Scale bar: 20 µm. The Pearson’s correlation was shown in bar graph from three independent experiments **(F)** Representative image of the spheroids was photographed and quantitative data analysis of spheroids numbers. Scale bars: 20 µm **(G)** The protein levels of stemness markers (CD133, Bmi-1, SOX-2) was examined by western blotting. The data were shown as the mean ± SD of three replicates. *p < 0.05, **p < 0.01, ***p < 0.001 vs. the control.

Celastrol significantly inhibited the viability of 786-O cells in a dose- and time-dependent manner (Figure 3A). In addition, celastrol reduced lipid accumulation in 786-O cells in a dose-dependent manner (Figure 3B). TC, free cholesterol (FC), and cholesterol ester (CE) were also decreased in celastrol-treated 786-O cells (Figure 3C). The inhibitory effect of celastrol on lipid accumulation was consistent with that of atorvastatin. Celastrol significantly decreased the protein expression of CAV-1 and LOX-1, while did not change flotillin-1 (FLOT-1) expression, a marker of lipid rafts (Figure 3D). Furthermore, celastrol inhibited the localization of CAV-1 and LOX-1 (Figure 3E). In addition, celastrol reduced the number of tumor spheres (Figure 3F). Celastrol also downregulated the levels of stemness markers, such as clusters of differentiation 133 (CD133), B-cell-specific Moloney murine leukemia virus insertion site 1 (Bmi-1), SRY-box 2 (SOX-2) in 786-O cells (Figure 3G). Collectively, these findings suggested that celastrol reduced intracellular lipid accumulation and impaired the stem cell properties, especially at 1 μΜ. Thus, 1 μΜ celastrol was chosen for subsequent experiments.

### Celastrol Impeded Lipid Accumulation and Impaired Stemness Properties Induced by ox-LDL *in vitro*.

**FIGURE 4:**
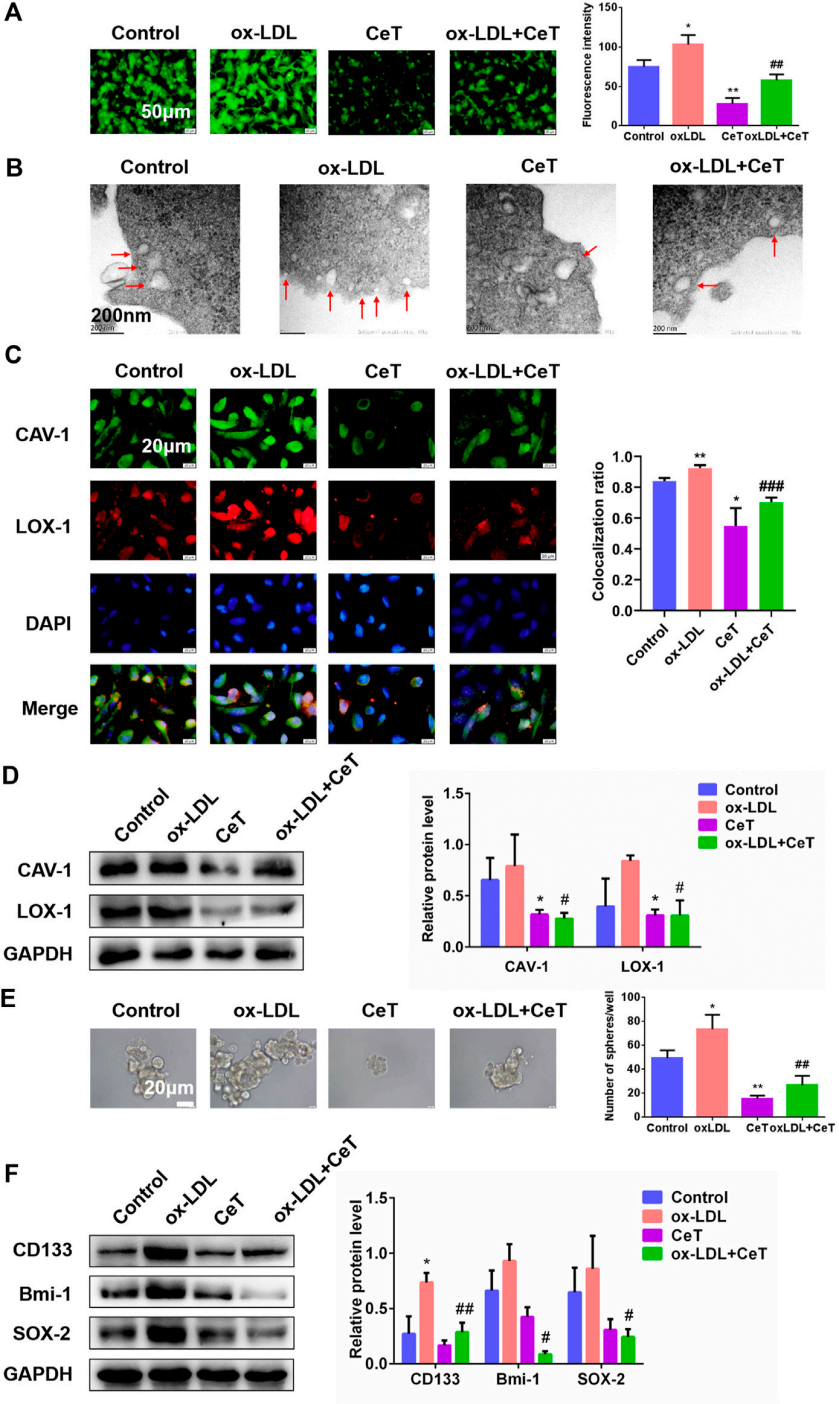
Celastrol repressed lipid accumulation and stemness properties induced by ox-LDL **(A)** The BODIPY fluorescence intensity of 786-O cells in the absence and presence of ox-LDL or CeT was observed by using fluorescent microscope. Scale bar: 50 μm **(B)** TEM showed the formation of caveolae in 786-O cells (red arrows). Scale bar: 200 nm **(C)** Co-localization of CAV-1 and LOX-1 in 786-O cells treated with ox-LDL or CeT. Scale bar: 20 µm. The Pearson’s correlation was shown in bar graph from three independent experiments **(D)** The expression of CAV-1 and LOX-1 in 786-O cells was detected by western blotting **(E)** Representative image and quantification of tumor spheres produced by 786-O cells with or without CeT and ox-LDL. Scale bar: 20 µm **(F)** Western blotting was performed to determine the expression of CD133, Bmi-1, SOX-2. The data were shown as the mean ± SD of three replicates. ^*^ *p* < 0.05, **p < 0.01, ***p < 0.001 vs. the control group; ^#^ *p* < 0.05, ^##^ *p* < 0.01 vs. the CeT group.

LDL is the major carrier of cholesterol to tissues. Early findings indicated that native LDL increased cholesterol content during *in vitro* incubations by receptor-independent, fluid-phase pinocytosis (Kruth 2011). Instead, oxidized LDL (ox-LDL), a pathologically modified lipoprotein, induces pronounced accumulation of cholesterol *via* its receptors LOX-1. Elevated ox-LDL levels are usually positively correlated with an increased risk of carcinogenesis (González-Chavarría, Fernandez, Gutierrez, González-Horta, Sandoval, Cifuentes, Castillo, Cerro, Sanchez and Toledo 2018). Herein, celastrol inhibited cell proliferation induced by ox-LDL (Supplementary Figure S2A). In addition, celastrol attenuated lipid accumulation and reduced the levels of TC, CE, FC induced by ox-LDL (Figure 4A, Supplementary Figures S2B–D). To confirm the presence of caveolae in 786-O cells, we performed ultrastructural studies by using TEM. Ox-LDL enrichment resulted in higher caveolae number and more caveolin-enriched vesicles (Figure 4B). In contrast, celastrol reduced the number of caveolae and increased the size of caveolar structure (Figure 4B). Representative microscopic images showed that celastrol inhibited the co-localization of CAV-1 with LOX-1 promoted by ox-LDL (Figure 4C). Celastrol also significantly decreased the expression of CAV-1 and LOX-1 in 786-O cells exposed to ox-LDL (Figure 4D). In addition, the ability of 786-O cells to generate tumor spheres was enhanced and the protein expression of CD133, Bmi-1, SOX-2 were upregulated under the culture with ox-LDL (Figures 4E,F). After treatment with celastrol, the number of spheres and stemness markers were significantly decreased (Figures 4E,F). Overall, these data demonstrated that celastrol reversed stemness induced by ox-LDL through reducing lipid accumulation.

### Celastrol Inhibited Lipid Accumulation and Stemness Properties *via* CAV-1

**FIGURE 5:**
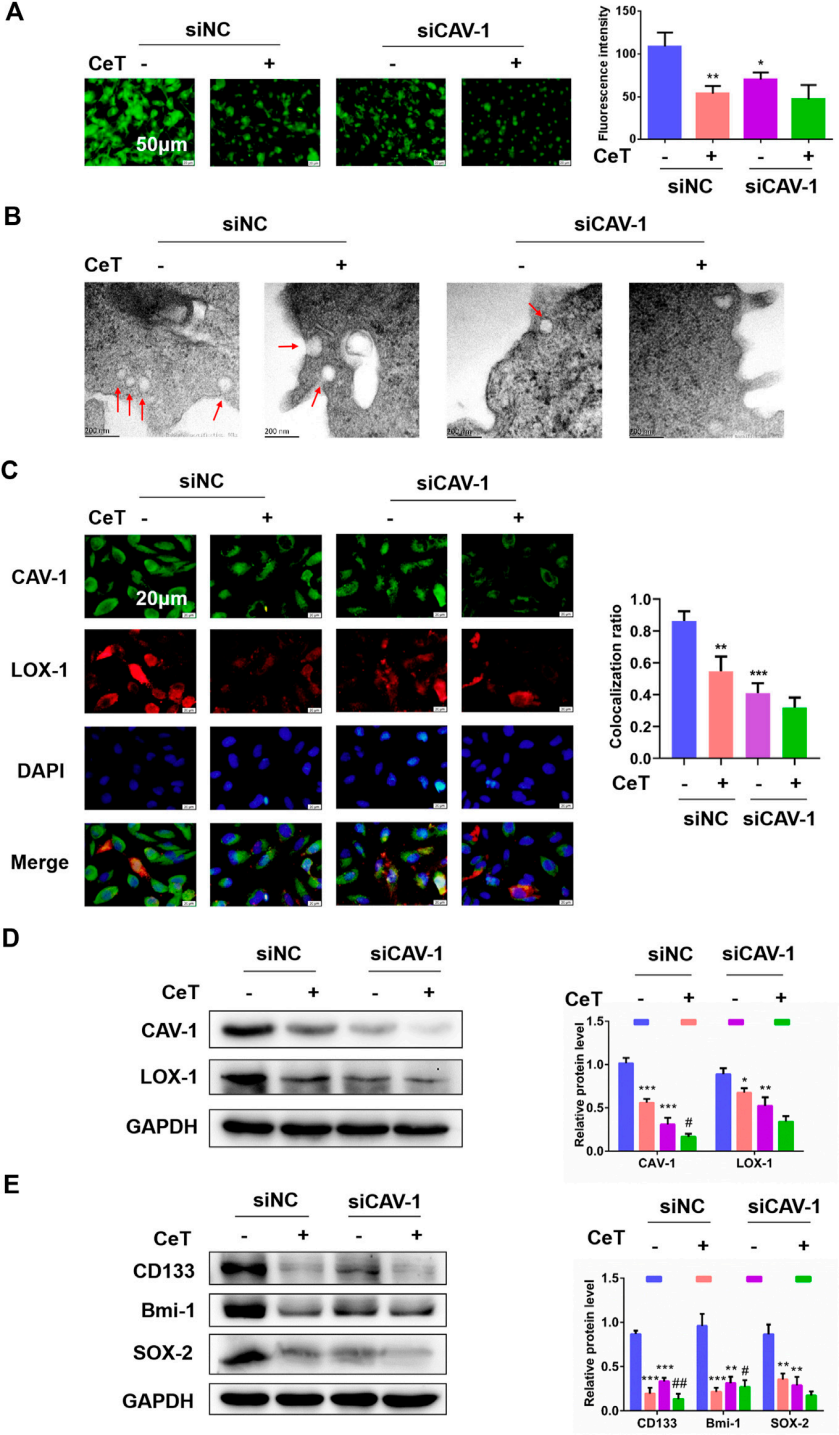
Celastrol impaired lipid accumulation and stemness *via* CAV-1 **(A)** The BODIPY fluorescence intensity was observed by using fluorescent microscope after knockdown of CAV-1. Scale bar: 50 μm **(B)** TEM assay confirmed that siNC cells displayed caveolae on the cellular surface, while few caveolae was present in siCAV-1 cells treated with CeT. Scale bar: 200 nm **(C)** Representative immunofluorescence staining showed the co-localization of CAV-1 and LOX-1 in 786-O cells transfected with siCAV-1. The Pearson’s correlation was shown in bar graph from three independent experiments **(D)** The levels of CAV-1 and LOX-1 were measured by western blotting **(E)** Western blotting of stemness-associated markers, including CD133, Bmi-1 and SOX-2. The data were shown as the mean ± SD of three replicates. *p < 0.05, **p < 0.01, ***p < 0.001 vs. the control; ^#^ *p* < 0.05, ^##^ *p* < 0.01 vs. the siCAV-1 cells.

We then examined whether celastrol inhibited lipid accumulation and prevents stemness properties *via* CAV-1. Firstly, 786-O cells were transfected with CAV-1-targeting siRNA (siCAV-1). As determined by western blotting, the expression of CAV-1 in three groups was more strongly decreased (Supplementary Figure S3A). Celastrol and siCAV-1 independently, significantly reduced lipid levels in general (Figure 5A). Treatment with celastrol on siCAV-1 cells significantly reduced FC and CE (Supplementary Figure S3B). Celastrol caused the loss of morphologically defined caveolae in siCAV-1 cells (Figure 5B). Furthermore, celastrol reduced the co-localization of CAV-1 and LOX-1 through inhibiting CAV-1 (Figure 5C). We also confirmed that celastrol reduced the protein expression of CAV-1 and LOX-1 in siNC cells or siCAV-1 cells (Figure 5D). Besides, silencing CAV-1 in 786-O cells impaired the potential for tumor sphere formation and the levels of stemness markers (Supplementary Figure S3C, Figure 5E). However, celastrol reduced sphere number and exhibited lower expression of stemness markers (Supplementary Figure S3C, Figure 5E). The celastrol treatment was more effective when combined with siCAV-1 as CAV-1 expression was reduced. These results demonstrated the synergistic effect of celastrol and siCAV-1 on lipid accumulation and stemness features.

### Celastrol functioned as a potent CAV-1 and LOX-1 interaction inhibitor.

**FIGURE 6:**
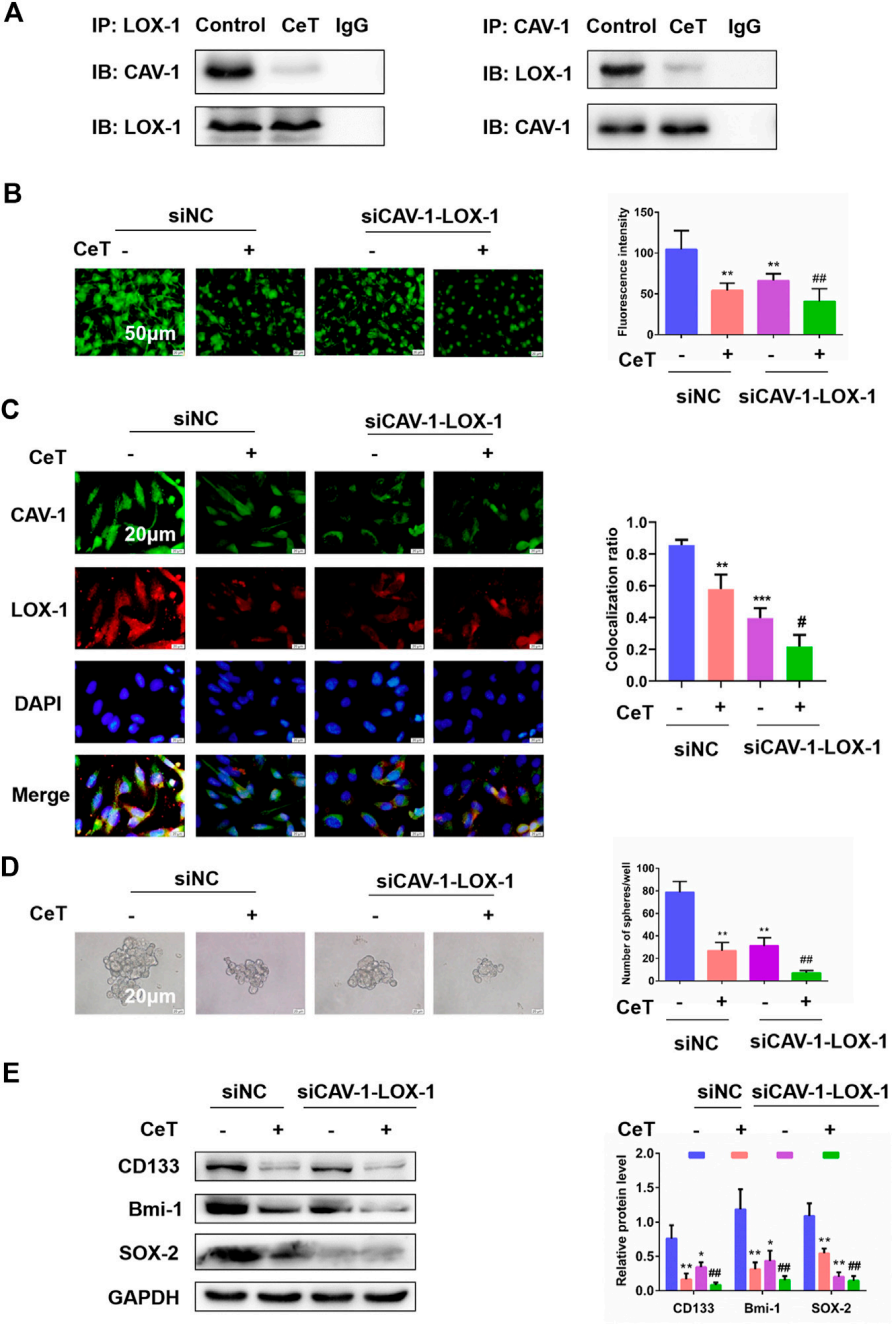
Celastrol attenuated lipid accumulation and stemness by inhibiting the binding of CAV-1 and LOX-1 **(A)** The specific interaction between CAV-1 and LOX-1 in untreated or CeT-treated 786-O cells was detected by co-immunoprecipitation test **(B)** BODIPY staining showed intracellular lipid levels. Scale bar: 50 µm **(C)** Co-localization of CAV-1 and LOX-1, as visualized at the subcellular level by double immunofluorescence staining. Scale bar: 20 µm. The Pearson’s correlation was shown in bar graph from three independent experiments **(D)** Self-renewal ability in siCAV-1-LOX-1 786-O cells was detected by sphere formation assays. Scale bar: 20 µm **(E)** Western blotting of stemness-associated markers, including CD133, Bmi-1 and SOX-2. *p < 0.05, **p < 0.01, ***p < 0.001 vs. the control group; ^#^ *p* < 0.05, ^##^ *p* < 0.01 vs. the siCAV-1-LOX-1 cells.

We performed co-IP analysis for CAV-1 and LOX-1 in 786-O cells treated with or without celastrol. Indeed, celastrol prevented the binding of CAV-1 and LOX-1 (Figure 6A). Then, we tested whether double knockdown of CAV-1 and LOX-1 enhanced the anti-cancer activity of celastrol in 786-O cells. 786-O cells were transfected with three different siRNAs against LOX-1. The siLOX-1-03 showed the best interference efficiency (Supplementary Figure S4A). Furthermore, compared with knockdown CAV-1 or LOX-1 alone, double knockdown of CAV-1 and LOX-1 showed better interference efficiency (Supplementary Figure S4B). After double knockdown of CAV-1 and LOX-1 in 786-O cells, celastrol further remarkably inhibited lipid accumulation and TC, FC, CE levels (Figure 6B, Supplementary Figure S4C). Immunofluorescence staining further confirmed that celastrol inhibited co-localization of CAV-1 and LOX-1 in double knockdown of CAV-1 and LOX-1 cells (Figure 6C). In addition, celastrol attenuated the stem-like properties and stemness markers expression after knockdown of CAV-1 and LOX-1 (Figures 6D,E). These results suggested that celastrol exerted its inhibitory activity on lipid accumulation and stemness by suppressing the binding of CAV-1 and LOX-1.

### Celastrol Suppressed Stem Cell-like Properties by Blocking Wnt/β-catenin Signaling Pathway

**FIGURE 7:**
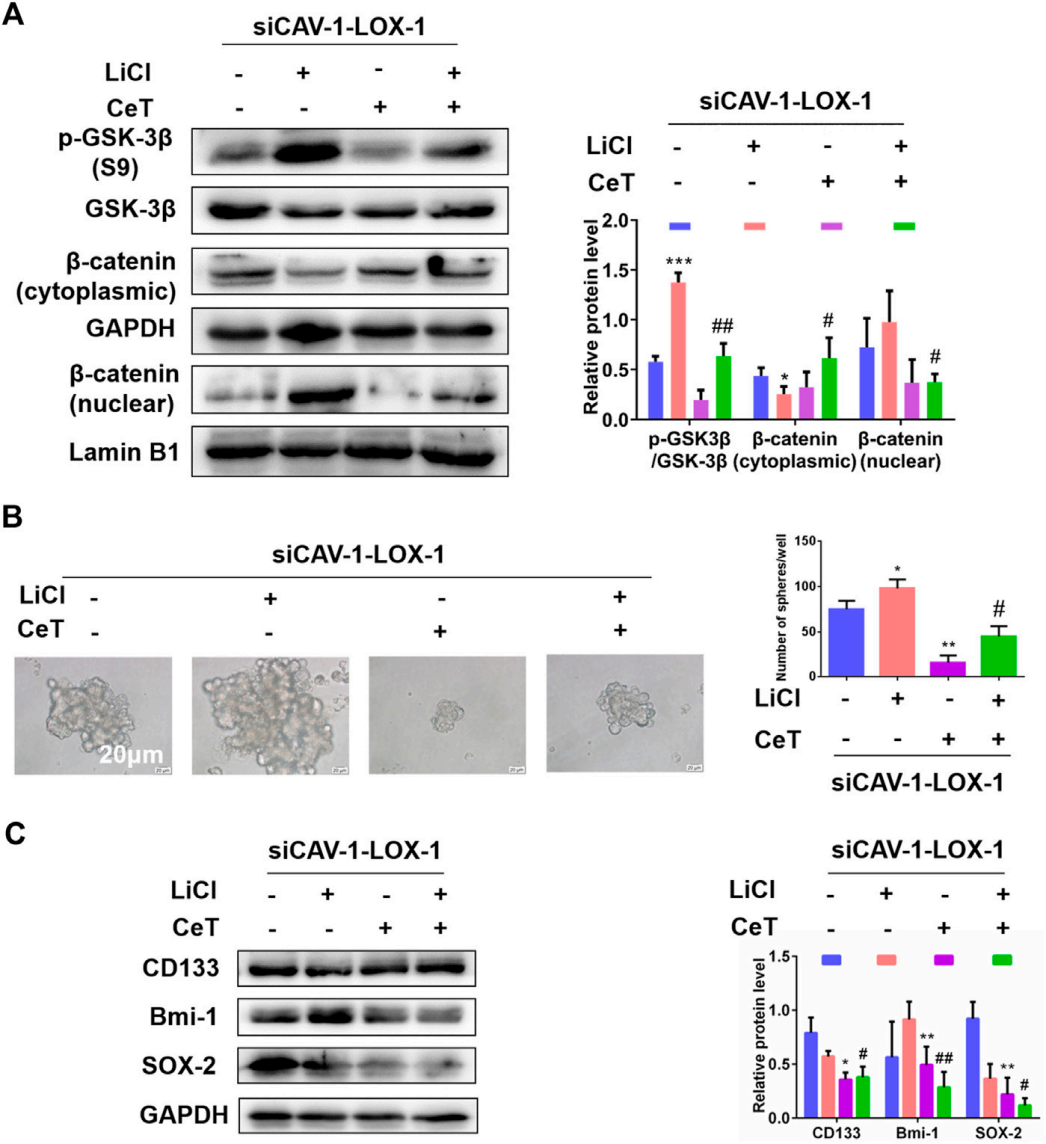
Wnt/β-catenin pathway was involved in stemness and lipid accumulation mediated by celastrol **(A)** In the absence and presence of LiCl, the protein levels of *p*-GSK-3β (S9), GSK-3β, β-catenin were examined by western blotting **(B)** Sphere formation capacity was measured by sphere formation assay. Scale bar: 20 µm **(C)** The expression of stemness related proteins was detected by western blotting. The data were shown as the mean ± SD of three replicates. ^*^ *p* < 0.05, **p < 0.01, ***p < 0.001 vs. the control group; ^#^ *p* < 0.05, ^##^ *p* < 0.01 vs. siCAV-1-LOX-1 cells treated with LiCl.

Under this premise, we next explored whether celastrol exerted anticancer effect by inhibiting CAV-1/LOX-1 and then blocking Wnt/β-catenin pathway. Lithium chloride (LiCl) was used as an activator of the Wnt/β-catenin pathway. The siCAV-1-LOX-1 cells were treated with Wnt/β-catenin pathway activator LiCl or celastrol, respectively. LiCl increased the protein level of the phosphorylation of glycogen synthase kinase-3β (p-GSK-3β) at serine 9 and promoted nuclear translocation of β-catenin (Figure 7A). In contrast, celastrol caused a decrease of p-GSK-3β (S9) and an increase of β-catenin level in the cytoplasm. We next determined the role of celastrol on stemness mediated by LiCl. As expected, celastrol inhibited sphere formation ability induced by LiCl (Figure 7B). When treated with celastrol, the expression of stemness markers induced by LiCl were reduced (Figure 7C). Interestingly, we observed that LiCl contributed to lipid accumulation and resulted in an increment of TC, FC, and CE levels, and these effects were reversed by celastrol (Supplementary Figures S5A,B). Based on the above findings, we concluded that Wnt/β-catenin signaling pathway is involved in the inhibitory effect of celastrol on the stemness-like properties of the 786-O cells.

## Disscusion

In recent years, increasing evidence has indicated that alterations in the lipid profile are associated with the occurrence, progression, and prognosis of various cancers, including RCC (Michela et al., 2015). The present study demonstrated that patients with reduced HDL level and elevated levels of TG, TC, LDL had a higher risk of ccRCC. This is consistent with a previous report that abnormal lipid profile can affect the risk of RCC(Zhang et al., 2013; Haddad et al., 2015; Kim et al., 2019).

CAV-1 was identified as a potential biomarker of ccRCC by using RNA sequencing. However, whether CAV-1 functions as an oncogene or a tumor suppressor during cancer progression is still controversial. The human CAV-1 gene locates at chromosome 7q31.1, and the incidence of tumor suppressor gene loss in high in a broad range of tumor types (Hurlstone et al., 1999). CAV-1 is down-regulated in breast cancer, pancreatic cancer, cervical cancer, and lung adenocarcinoma (Chiu et al., 2011; Chen et al., 2013; Zhang et al., 2017; Volonte et al., 2018). In contrast, the expression of CAV-1 was increased in ccRCC, prostate cancer, bladder cancer, and hepatocellular carcinoma (Thomas et al., 2011; Ruan et al., 2017; Lin et al., 2019; Mao, Tey, Ko, Kwong, Gao, Ng, Cheung, Guan and Yam 2019). More meaningfully, the upregulation of CAV-1 is related to advanced tumor stage, lymph node metastasis and poor prognosis of cancer patients (Kato et al., 2020). Thus, CAV-1 may imply that CAV-1 acts as a tumor promoter. In the initial stages of tumor progression, tissue undergo oncogenic transformation. The decreased expression of CAV-1 promotes the rapid proliferation of cancer cells. In the later stages, with the larger tumor and malignant progression, cancer cells need to adapt to the complex microenvironment (Fu et al., 2017). This may be a plausible explanation for the increased expression of CAV-1 in the aggressive forms and metastatic ccRCC rather than the less aggressive forms.

Based on the microarray results, CAV-1 might be one of the lipid metabolism related genes regulated by celastrol in ccRCC. It is generally believed that CAV-1 is essential and sufficient for caveolae formation. However, previous studies (Sohn et al., 2018) and our investigation suggest that formation of stable caveolae may depend on the interaction between cholesterol and CAV-1 protein. Previous studies have found that the caveolae is a dynamic structure. They can bud from the plasma membrane, and their internalization is regulated by the molecular transport machinery of vesicle budding, docking, and fusion (Westermann et al., 1999). Herein, we found that ox-LDL enrichment resulted in a large number of caveolin-enriched vesicles in ccRCC cells. The observations support the important role of lipids in the formation of caveolar structure. In this case, the structural damage caused by celastrol may be the reason for the increase in the size of the caveolar structure. Therefore, it is also possible that celastrol functions as an inhibitor of CAV-1 protein and caveolae number in ccRCC cells.

Hirsh et al. (Hirsch et al., 2010) described the high expression of LOX-1 in pathologically advanced breast and prostate carcer. We demonstrated that LOX-1 was important for maintaining lipid storage and stemness in ccRCC cell lines. But so far, there are no studies on the effect of CAV-1/LOX-1 on cancer development. Celastrol led to an indirect effect on lowering intracellular lipid level, which was derived from inhibiting the binding of CAV-1 and LOX-1The reduction is not only related to variation in the number of caveolae, but also the impairment of their functions. These studies are certainly indirect, and it will be imperative for future studies to inhibit CAV-1 and LOX-1 by a direct interaction with the C type lectin domain (CTLD) recognition domain to indicate the previously unknown and novel pleiotropic effect of celastrol.

Recent evidence has showed that Wnt/β-catenin pathway played a critical role in the stemness phenotype of ccRCC(Lu et al., 2017). In canonical Wnt signaling, the extracellular ligand Wnt leads to inactivation of GSK-3β, resulting in dephosphorylation and stabilization of β-catenin protein in the cytoplasm. Increased level of β-catenin promotes its nuclear translocation, and its interaction with transcription factors LEF/TCF (lymphoid enhancer factor/T cell factor) activate the expression of target genes such as SOX-2, Nanog and Oct4, thereby maintaining stemness and inducing tumorigenesis (Holland et al., 2013). Remarkably, GSK-3β activity is increased by the site-specific phosphorylation of Tyr216, whereas the phosphorylation of Ser9 inhibits GSK-3β activity (Rayasam et al., 2009). In addition, several evidence suggest that CAV-1 may function as a regulator of self-renewal signaling pathways in stem cells (Bai et al., 2014).

However, our study had some unavoidable limitations. First, ccRCC might cause dyslipidemia, and its underlying mechanisms also include effects on cholesterol absorption, transport, metabolism, or utilization. Previous study has supported the view that cancers might affect cholesterol metabolism or utilization due to plasma cholesterol levels reverted to normal after cancer remission (Yun et al., 2019). Second, it is worth considering other confounding factors, such as variety of food intake and dyslipidemia, because these factors could also increase the risk of cancer (Morel et al., 2019). In addition, the candidates selected for the clinical samples in the present study were all Chinese. Thus, our results might not necessarily apply to other races or ethnicities. Considering the high prevalence of dyslipidemia in developed countries, further studies on the potential role of serum lipids as the etiologic factor of ccRCC will be of substantial importance for public health.

## Conclusion

**FIGURE 8:**
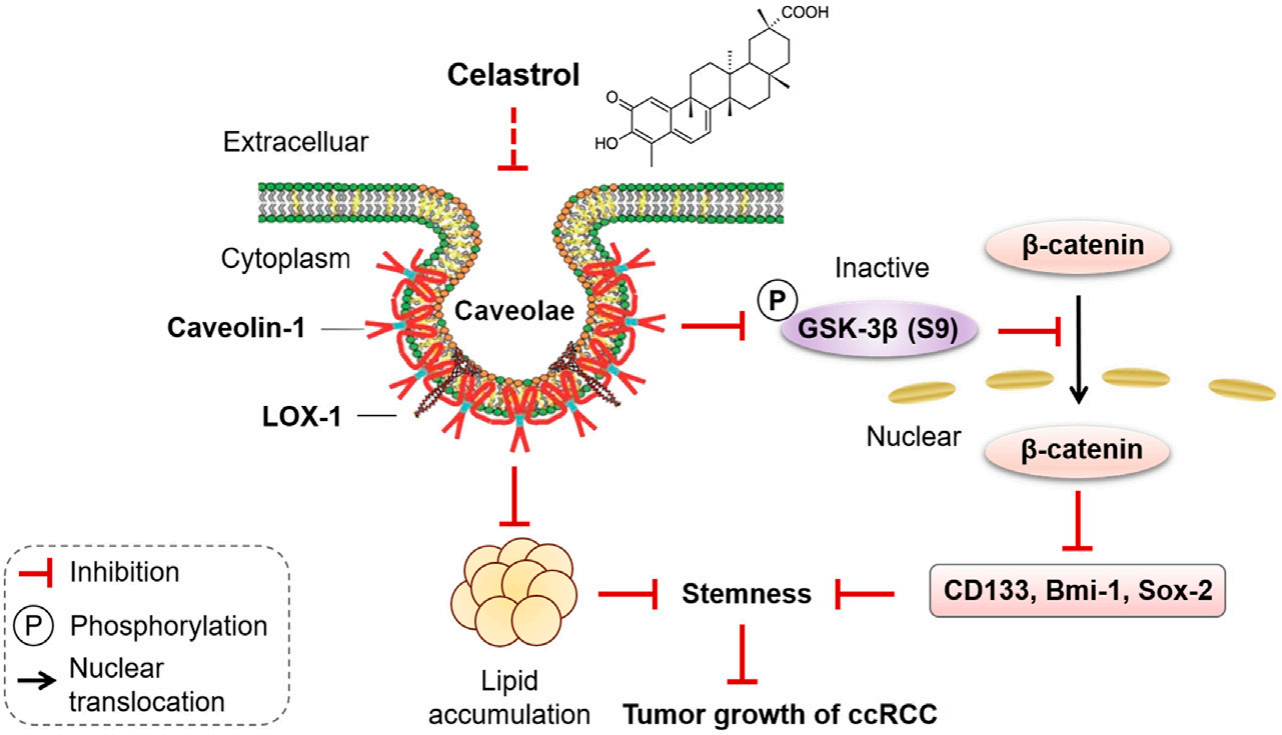
Diagram summarized the effect of celastrol on lipid accumulation and stemness in ccRCC progression. Celastrol, as an active compound of traditional Chinese medicine, inhibited the binding of CAV-1 and LOX-1, reduced lipid accumulation, blocked the phosphorylation of GSK-3β at Ser9, prevented nuclear translocation of β-catenin, thereby impairing stem-like properties, eventually inhibited tumor growth of ccRCC.

In conclusion, celastrol effectively attenuated lipid accumulation through inhibiting the interaction of CAV-1 and LOX-1, and then blocked Wnt/β-catenin signaling pathway to impair stem-like properties, thereby suppressed tumor growth of ccRCC (Figure 8). The present study provides evidence for the development of celastrol as a promising anticancer agent.

## Data Availability

The datasets presented in this study can be found in online repositories. The name of the repository and accession numbers are as follows: https://www.ncbi.nlm.nih.gov/sra (PRJNA705508, PRJNA705508, SAMN18090835, SAMN18090836, SAMN18090837).
