# Supplementary material for: Celastrol Attenuates Lipid Accumulation and Stemness of Clear Cell Renal Cell Carcinoma via CAV-1/LOX-1 Pathway
Source: Front Pharmacol. 2021 Apr 16;12:658092. doi: 10.3389/fphar.2021.658092 (PMC8085775; doi:10.3389/fphar.2021.658092)
Supplement: Supplementary file 1 [file datasheet1.docx]

Supplementary Material

# Supplementary Figure


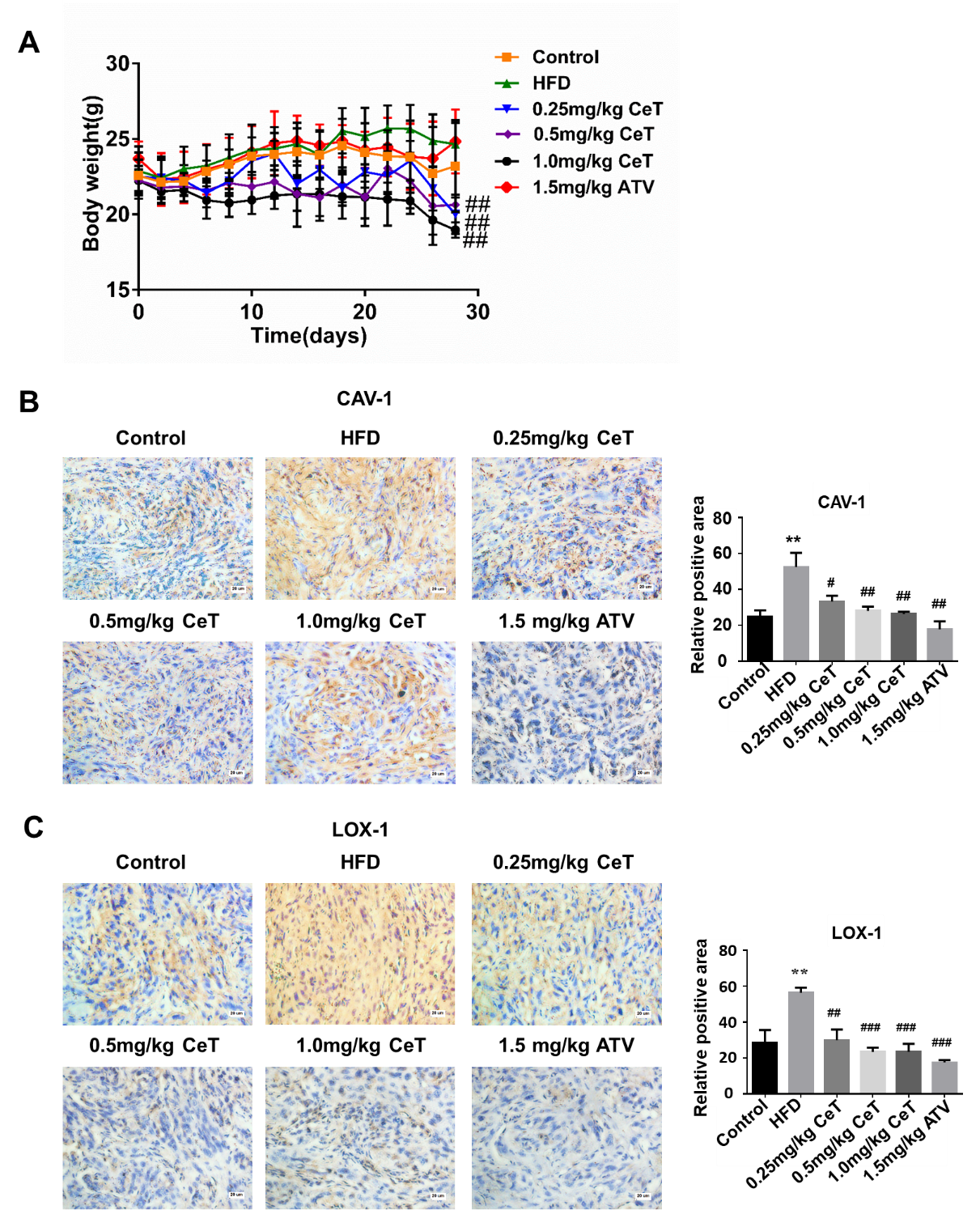

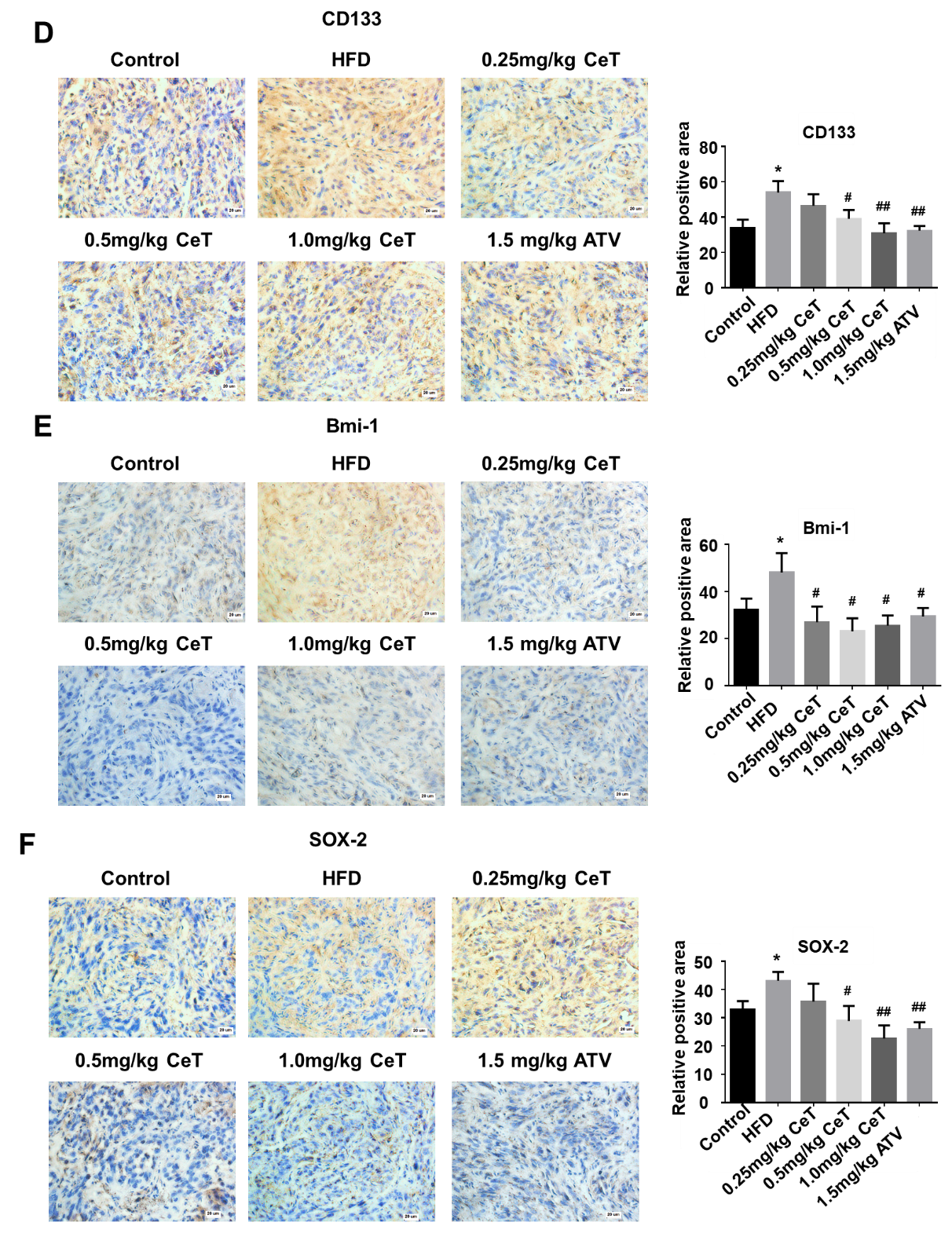


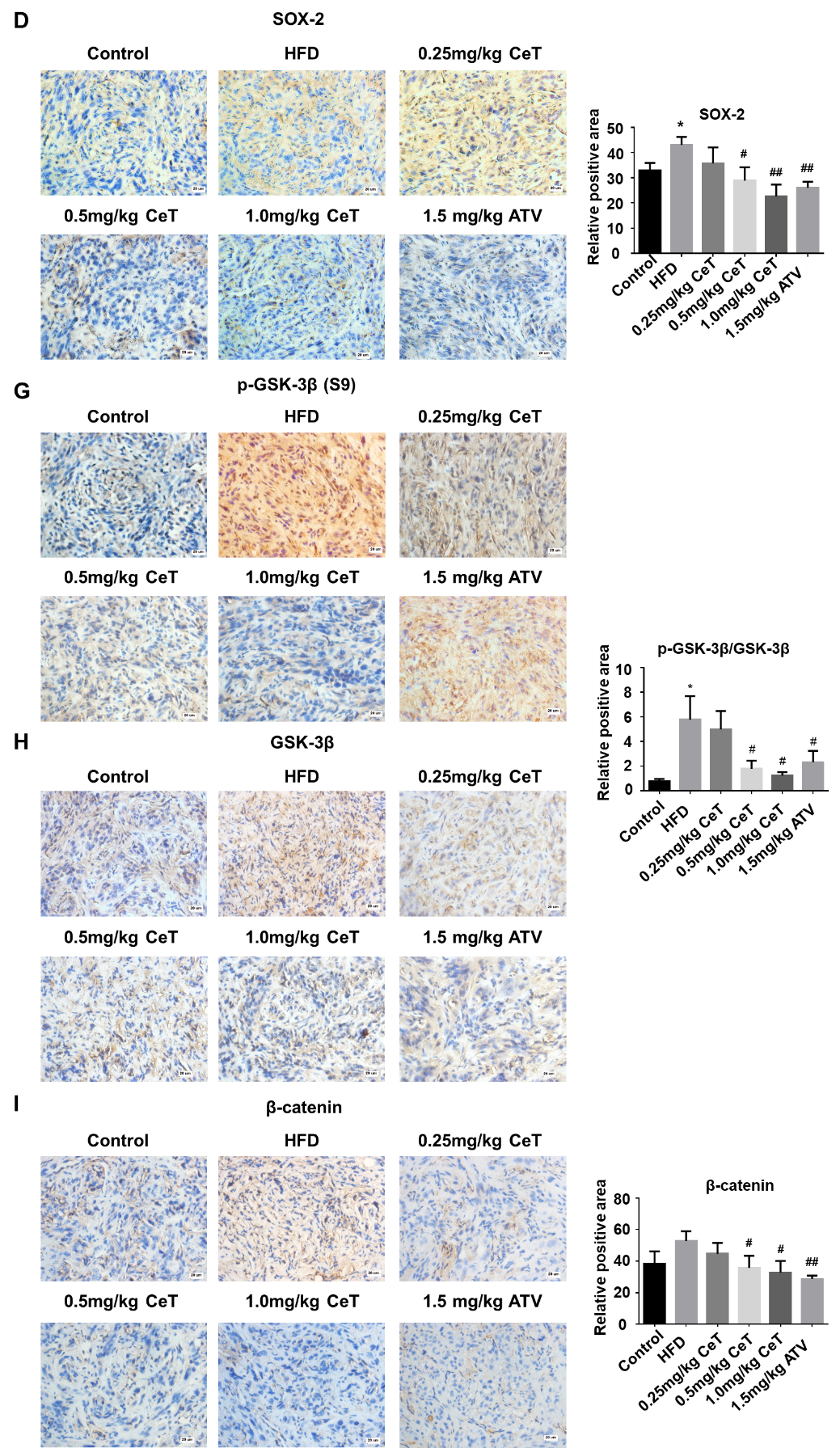


**Supplementary Figure 1.** **(A)** Body weight at the indicated time-points were calculated every two days after treatment. **(B-I)** Immunohistochemistry analyses of CAV-1, LOX-1, CD133, Bmi-1, SOX-2, p-GSK-3β (S9), GSK-3β, and β-catenin in tumor tissues. Scale bar: 20 μm. ^*^*P*<0.05, ^**^*P*<0.01 vs. the control group; ^#^*P*<0.05, ^##^*P*<0.01, ^###^*P*<0.001 vs. the HFD group.


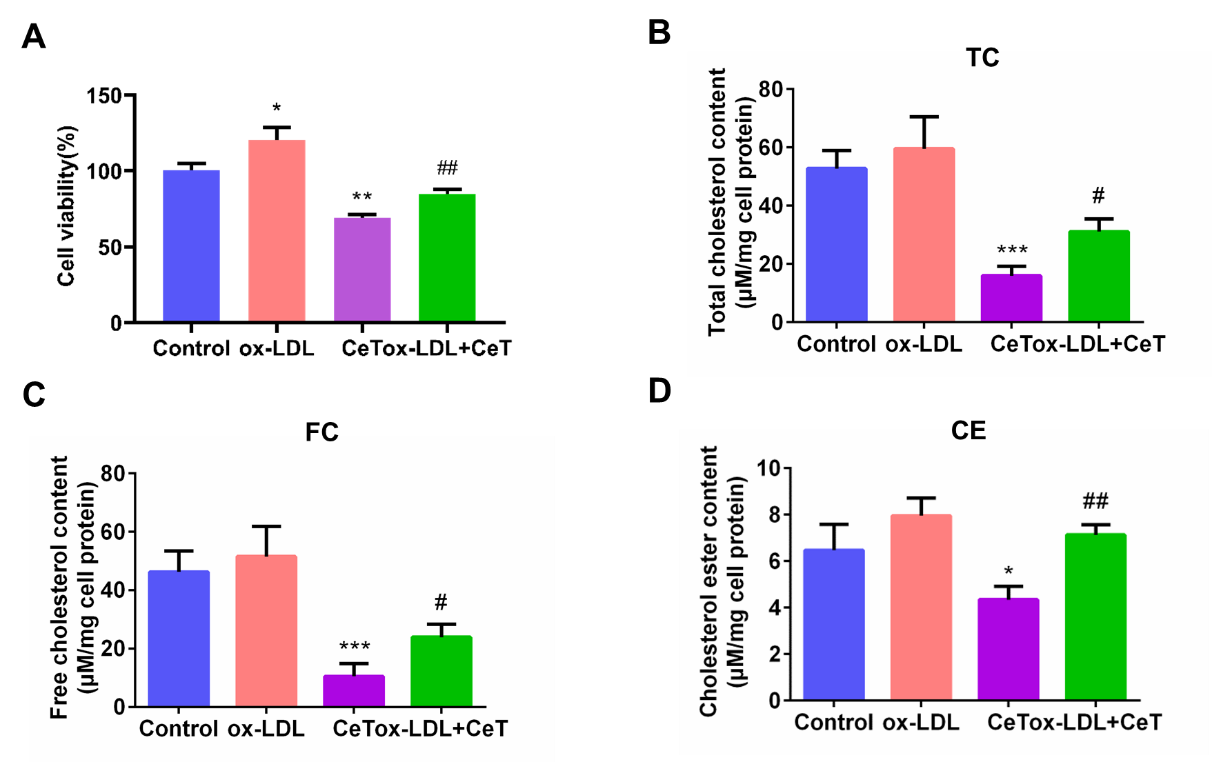


**Supplementary Figure 2.** **(A)** Cell viability was detected by CCK-8 assay. **(B-D)** The effect of CeT and ox-LDL on the levels of TC, FC, and CE was examined by using cholesterol enzyme assay. ^*^*P*<0.05, ^**^*P*<0.01, ^***^*P*<0.001 vs. the control group; ^#^*P*<0.05, ^##^*P*<0.01 vs. the CeT group.


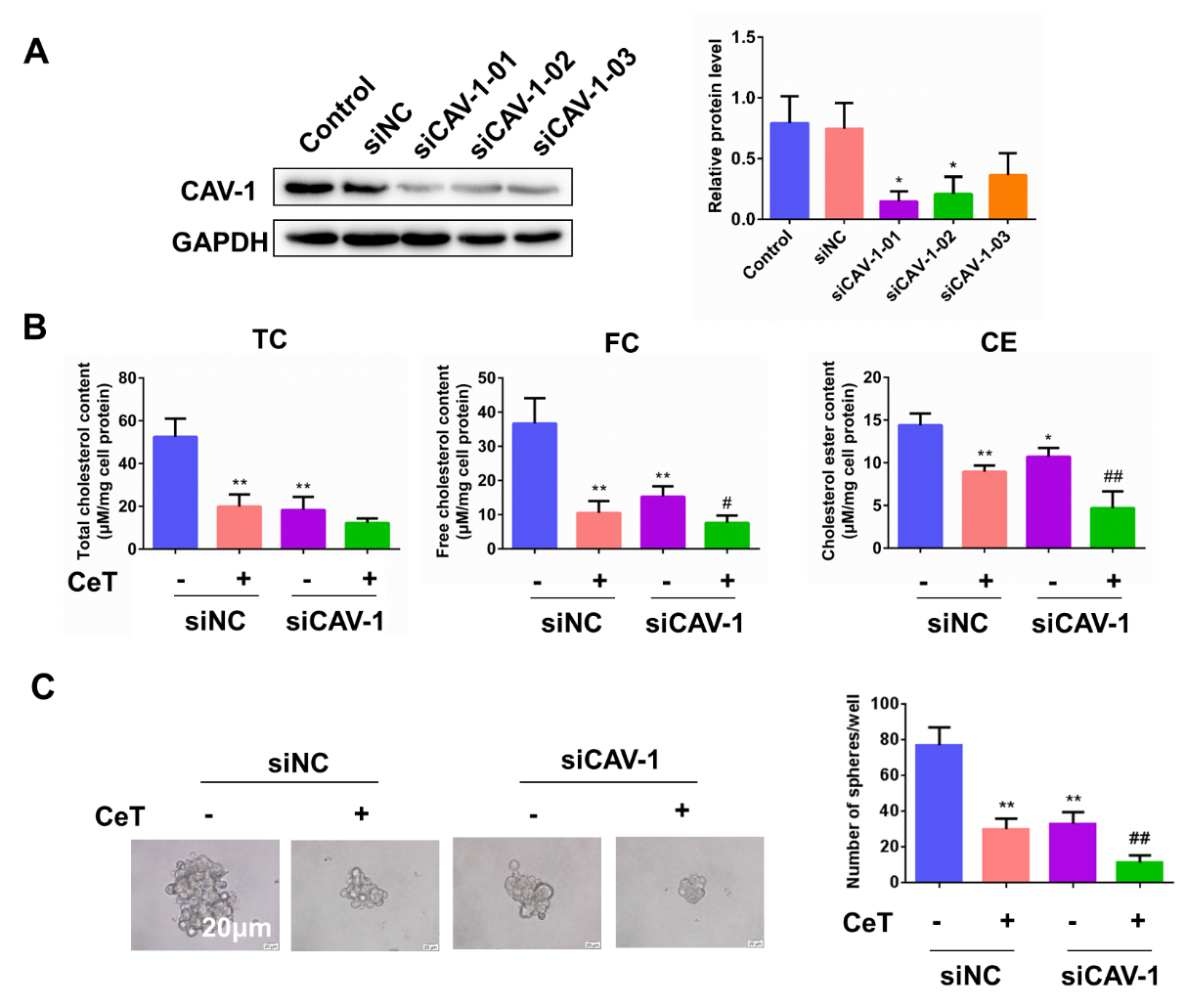


**Supplementary Figure 3.** **(A)** 786-O cells were transfected with siRNA against CAV-1 (siCAV-1). The cells in control group were transfected with negative control siRNA (siNC). Western blotting was applied to determine knockdown efficiency. **(B)** TC, FC, and CE levels was checked by using cholesterol enzyme assay. **(C)** Sphere formation ability of 786-O cells transfected with siCAV-1 was examined (left panel) and quantified (right panel). Scale bar: 20 µm. ^*^*P*<0.05, ^**^*P*<0.01 vs. the control; ^#^*P*<0.05, ^##^*P*<0.01 vs. the siCAV-1 cells.


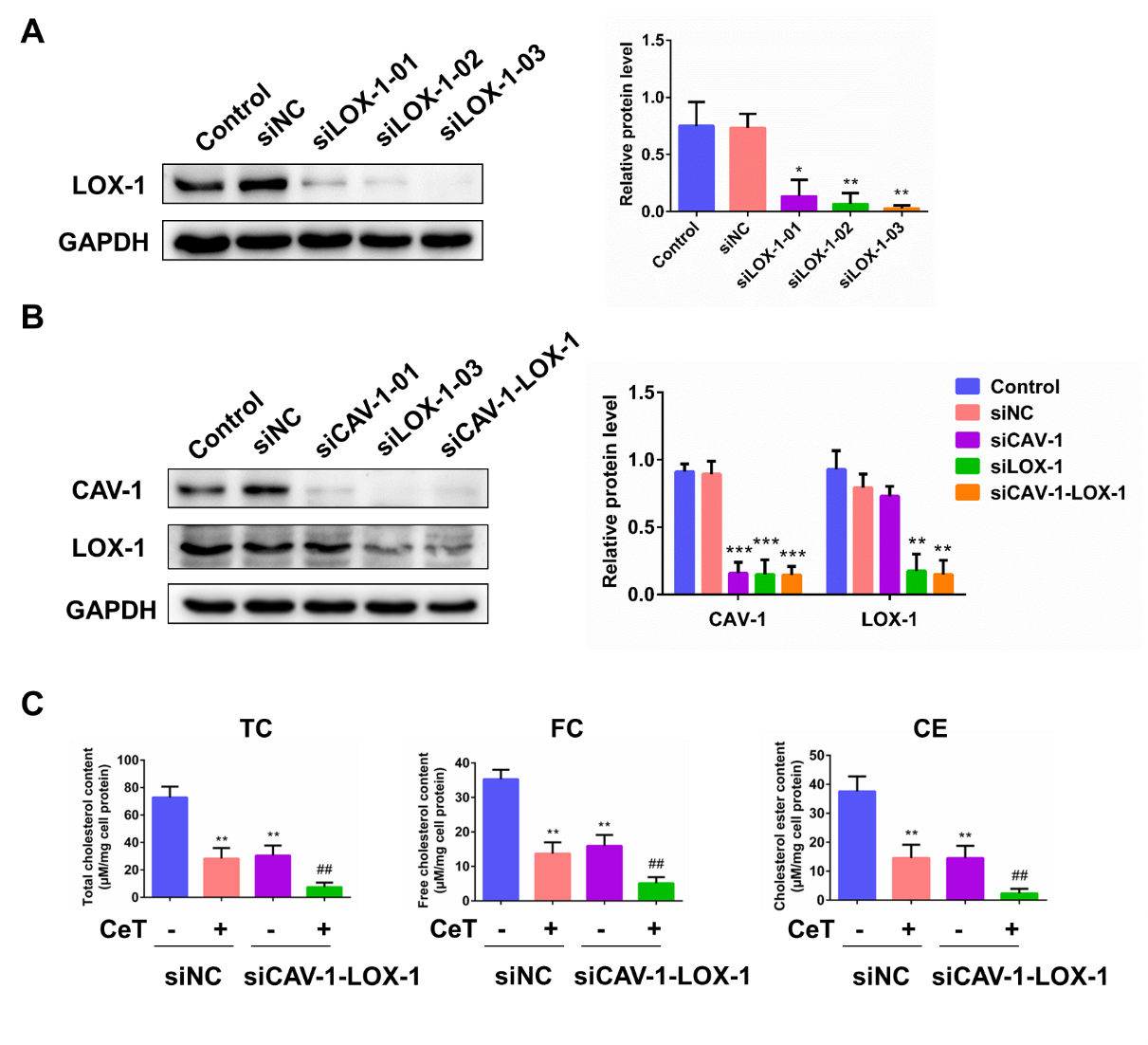


**Supplementary Figure 4.** **(A)** Confirmation of LOX-1 knocking down in 786-O cells by western blotting. **(B)** Detection of the expression of CAV-1 and LOX-1 in 786-O cells treated with siRNA targeting CAV-1 and/or LOX-1 by western blotting, respectively. **(C)** The levels of TC, FC, CE were measured by cholesterol enzyme assay. ^*^*P*<0.05, ^**^*P*<0.01, ^***^*P*<0.001 vs. the control group; ^##^*P*<0.01 vs. the siCAV-1-LOX-1 cells.


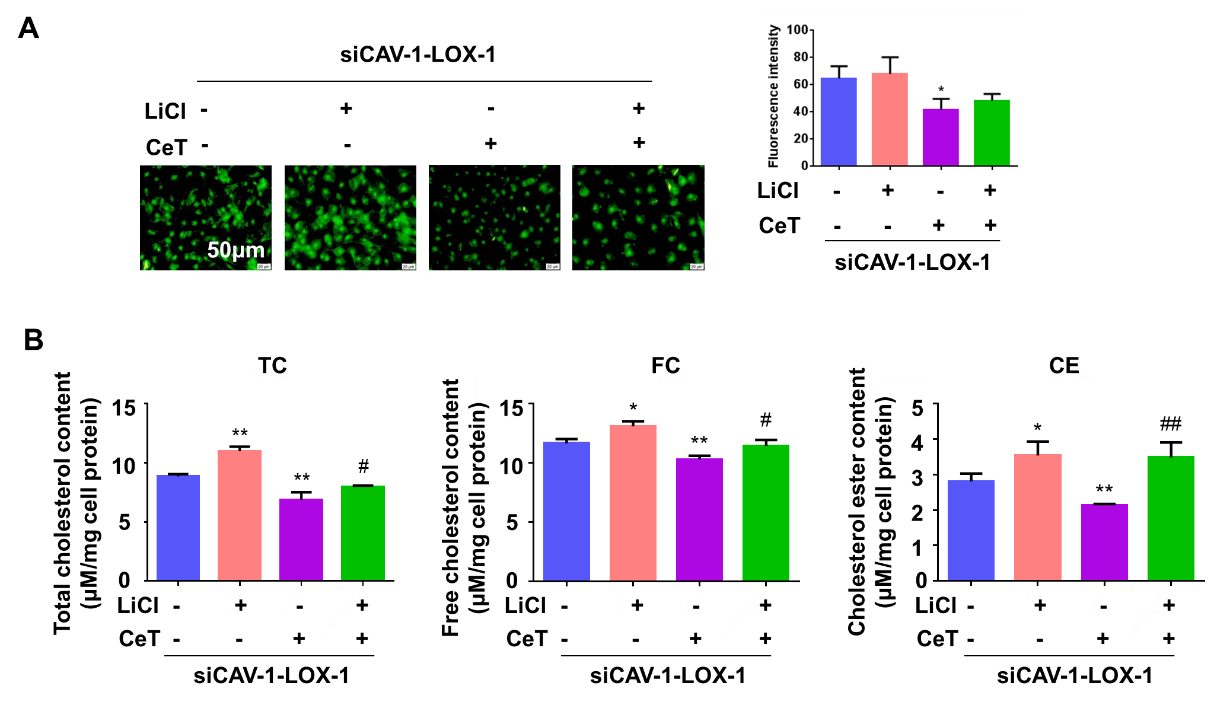


**Supplementary Figure 5.** **(A)** BODIPY 493/503 staining showed the effects of CeT and LiCl on lipid droplets in siCAV-1-LOX-1 cells. The fluorescence intensity was quantified and displayed on the right. Scale bar: 50 µm. **(B)** The levels of TC, FC, and CE were detected by using cholesterol enzyme assay. ^*^*P*<0.05, ^**^*P*<0.01 vs. the control group; ^#^*P*<0.05, ^##^*P*<0.01 vs. siCAV-1-LOX-1 cells treated with LiCl.

# Supplementary Table

Table 1 Clinicopathological characteristics of ccRCC patients

| Characteristics | | Cases | Patients (%) | Characteristics | | Cases | Patients (%) |
| --- | --- | --- | --- | --- | --- | --- | --- |
| Age  (years) | <53 | 172 | 47.5 | Lymph node metastasis | | 29 | 8.0 |
|  | ≥53 | 190 | 52.5 | Distant metastasis | | 27 | 7.5 |
| Gender | Male | 245 | 67.7 | HDL (mmol/L) | <1.04 | 168 | 46.4 |
|  | Female | 117 | 32.3 |  | 1.04-1.55 | 60 | 16.6 |
| Tumor size (cm) | >3 | 142 | 39.2 |  | >1.55 | 24 | 6.6 |
|  | ≤3 | 55 | 15.2 | TG (mmol/L) | 0.22-1.21 | 36 | 9.9 |
| Tumor number | >1 | 42 | 11.6 |  | >1.21 | 180 | 49.7 |
|  | =1 | 146 | 40.3 | TC (mmol/L) | <2.86 | 36 | 9.9 |
| T stage | T1+T2 | 42 | 11.6 |  | 2.86-5.89 | 84 | 23.2 |
|  | T3+T4 | 6 | 1.66 |  | >5.89 | 60 | 16.6 |
| N stage | N0 | 40 | 11.0 | LDL (mmol/L) | 0-3.4 | 72 | 19.9 |
|  | N1 | 5 | 1.38 |  | >3.4 | 108 | 29.8 |
| M stage | M0 | 42 | 11.6 |  |  |  |  |
|  | M1 | 5 | 1.38 |  |  |  |  |

ccRCC: Clear cell renal cell carcinoma; HDL: high density lipoprotein; TG: triglyceride; TC: total cholesterol; LDL: low density lipoprotein.
